# How Epigenetic Clocks Tick: Unpacking the Black Box by Deciphering Biological Pathways and Downstream Transcriptomic Signatures of Accelerated Aging

**DOI:** 10.1093/geroni/igaf122.1246

**Published:** 2025-12-31

**Authors:** Thalida Arpawong, Eileen Crimmins

**Affiliations:** University of Southern California, Los Angeles, California, United States; University of Southern California, Los Angeles, California, United States

## Abstract

Epigenetic clocks derived from DNA methylation data are often used to assess biological aging and predict health outcomes and mortality. Several epigenetic clocks have been developed to date, although the basis for their differential predictive power on health outcomes remains unclear due to the “black box” nature of the clocks and lack of information about the specific biological processes they assess. DNA methylation is biologically significant due to its role in regulating gene expression, but the degree to which these clocks reflect biological gene expression pathways as well as common or unique biological processes is not well understood. We utilized data from 3,227 individuals in the US Health and Retirement Study, measuring gene expression via RNA sequencing and epigenetics through DNA methylation. Epigenetic clocks including the Horvath, Hannum, PhenoAge, GrimAge, and DunedinPACE clocks, were used as independent variables in an 80% training sample (n = 2,584) to conduct differential gene expression analyses. Differentially expressed genes were identified for each clock, then functionally annotated to identify shared and unique biological processes and pathways underlying each clock. In a 20% hold-out test sample (n = 645), we evaluated transcriptomic signatures, derived from differentially expressed genes from each clock, alongside the epigenetic clocks for their association with mortality and multiple aging-related health outcomes. The results revealed more unique than common biological processes, shedding light on why each clock predicts different health and aging phenotypes with varying strength. Findings clarify the internal mechanisms of these clocks, offering biological insights and paving the way for translation into potential clinical applications aimed at predicting aging-related health trajectories.

